# 7.D. Workshop: Older adults’ resilience and vulnerability to COVID-19: 2-years results of multicentric LOST project

**DOI:** 10.1093/eurpub/ckac129.409

**Published:** 2022-10-25

**Authors:** Organised by: University of Pavia (Italy), Chair persons: Anna Odone (EUPHA-DH, EUPHA-IDC), Silvano Gallus (Italy)

## Abstract

COVID-19 pandemic and consequent mitigation and containment measures greatly impacted people's daily life. Older adults, especially those with chronic conditions, have paid the highest price for the emergency. Indeed, they are the most at-risk of severe and deadly forms of COVID-19; they deal with unmet healthcare needs because of considerable pressure on healthcare systems; they are at higher risk of worsening lifestyles, mental and socioeconomic status. In this context, we designed and are currently conducting the multi-partner project LOckdown and lifeSTyles in Lombardia (LOST in Lombardia), funded by AXA and Lombardy region. We will present the design and outputs of a telephone-based cross-sectional study conducted in the fall of 2020 on a representative sample of adults aged more than 65 years living in Lombardy. Researchers from the University of Pavia, Mario Negri Institute of Pharmacological Research, Vita-Salute San Raffaele University, Insubria University, University of Genoa, Bocconi University, Health Protection Agency of Bergamo and Brianza are involved. This project aims to evaluate the COVID-19 pandemic impact and nationwide stay-at-home order on a wide range of behavioural and health-related outcomes among the older population and provide useful evidence to plan, implement, and evaluate targeted prevention programmes and welfare policies facing pandemic consequences.

The workshop will be structured as follows:

We will first set the scene by focusing on the determinants of SARS-CoV-2 infection in the older adult population.

The second panellist will discuss changes in healthcare services use and delivery during the pandemic. Sociodemographic, physical and mental health-related determinants’ roles will be thoroughly analysed.

Data on the access to hospital in older adults with chronic conditions from two local health Units from the Lombardy region, Italy during the first wave of the pandemic wee also presented.

We will then provide two original contributions about lifestyle changes.

In the first one, we will present data exploring how Mediterranean diet adherence has changed. A Mediterranean COVID-19 Pandemic Score was computing by scoring modifications in the consumption of nine food groups and five diet-related behaviours, and predictors of favourable changes will be debated.

The fifth panellist will open up about the mental health burden. The prevalence of depression, anxiety, insomnia and hopelessness before and during the COVID-19 lockdown will be assessed, identifying subgroups at higher risk of mental distress due to pandemic-associated restrictions.

In the end, we will engage in a fruitful discussion with the audience on the data presented and practical public health implications, exploring how they can inform critical policy debates addressing health system resilience and health inequalities at the European and global levels.

**Key messages:**

• Public health management of older people and chronic patients represents a major challenge within and beyond pandemic times, as they have been disproportionally impacted by COVID-19.

• Evidence-based recommendations are needed for the post-pandemic phase, and the results on successes and failures from one of the worst-affected regions provide guidance for the rest of Europe.

